# Spatial Clustering and Local Risk Factors of Chronic Obstructive Pulmonary Disease (COPD)

**DOI:** 10.3390/ijerph121215014

**Published:** 2015-12-10

**Authors:** Ta-Chien Chan, Hsuan-Wen Wang, Tzu-Jung Tseng, Po-Huang Chiang

**Affiliations:** 1 Research Center for Humanities and Social Sciences, Academia Sinica, Taipei 115, Taiwan; dachianpig@gmail.com (T.-C.C.); j_lucy1002@yahoo.com.tw (T.-J.T.); 2 Division of Family Medicine, Fangliao General Hospital, Pingtung 940, Taiwan; jackysour@gmail.com; 3 Department of Public Health, College of Medicine, Fu Jen Catholic University, New Taipei 242, Taiwan; 4 Institute of Population Health Sciences, National Health Research Institutes, Zhunan 350, Taiwan; 5 Department of Public Health, China Medical University, Taichung 400, Taiwan

**Keywords:** COPD, SaTScan, smoking, aborigines, Taiwan

## Abstract

Chronic obstructive pulmonary disease (COPD) mortality has been steadily increasing in Taiwan since 2009. In order to understand where the hotspot areas are and what the local risk factors are, we integrated an ecological and a case-control study. We used a two-stage approach to identify hotspots and explore the possible risk factors for developing COPD. The first stage used the annual township COPD mortality from 2000 to 2012 and applied the retrospective space-time scan statistic to calculate the local relative risks in each township. In the second stage, we conducted a case-control study, recruiting 200 patients from one local hospital within the one identified hotspot area located in southern Taiwan. Logistic regression was applied for analyzing the personal risk factors of COPD. The univariate analyses showed that higher percentages of aborigines, patients with tuberculosis (TB) history, and those with smoking history had COPD (*p* < 0.05). After controlling for demographic variables, aboriginal status (adjusted odds ratios (AORs): 3.01, 95% CI: 1.52–5.93) and smoking history (AORs: 2.64, 95% CI: 1.46–4.76) were still the two significant risk factors. This two-stage approach might be beneficial to examine and cross-validate the findings from an aggregate to an individual scale, and can be easily extended to other chronic diseases.

## 1. Introduction

Population ageing is an ongoing trend for many developed and developing countries [1]. At the same time, chronic diseases also pose great disease burdens on the elderly. Among these, chronic obstructive pulmonary disease (COPD) was the fifth leading cause of death in 2002 throughout the World [2], and it is predicted to become the third leading cause of death in 2020 [3]. In Taiwan, COPD age-adjusted mortality rates for the whole male population during 1999–2007 declined from 26.83 to 19.67 per 100,000 population, and for females declined from 8.98 to 5.70 per 100,000 population [4]. However, we find a resurging trend of COPD mortality after 2009 in this study. Although the trend of mortality was declining from 1999 to 2007, the prevalence of COPD among adults aged 40 years and over is around 7.8%, and the resultant medical expenditures are still high, with an estimated annual total burden of COPD in Taiwan of around €200 million [5]. To alleviate the disease burden of COPD, it is worth finding out what risk factors may exist locally in order to reduce the incidence of COPD. There are many risk factors identified in previous studies, including personal risk factors such as smoking [6,7] and exposure to biomass smoke [8], or socio-economic factors such as lower income [9] and the rural and urban difference [10], as well as environmental factors such as air pollution [11,12] and medical accessibility [13,14]. In our previous ecological studies, we also identified the smoking rate, the percentage of aborigines, PM_10_ and altitude as positively correlated with COPD mortality [4]. Although we have found clusters and ecological correlations in southern Taiwan, we still do not know the exact local risk factors within the clusters.

Thus, we further extended mortality data to 2012 and used space-time scan statistics to help us identify spatio-temporal COPD clusters. Then, we initiated the hospital-based case-control study in the hotspot clustering area. This study design is unlike traditional ones which do either ecological analysis alone or statistical analysis from public health or clinical databases. The integration of these two designs can help filter out common risk factors and identify any hotspots in the first stage. Then, a local epidemiological survey might be beneficial to collect possible individual risk factors and understand local situations clearly in the second stage. 

The goals of this study were to try to identify any hotspot areas of COPD mortality and then further conduct a case-control study to identify local risk factors. It is suggested that this two-stage study design also be utilized for investigating other chronic diseases.

## 2. Materials and Methods

### 2.1. Ethics

This study was approved by the institutional review board (IRB) of Academia Sinica (IRB #: AS-IRB-BM 13057). The second-hand anonymous cause of death database was used for the spatial cluster analysis. Data from townships with fewer than three COPD deaths were omitted, in line with the regulations on using official health databases made by the Department of Statistics, Ministry of Health and Welfare. 

### 2.2. Data Collection

After we identified a hotspot area, we further conducted a hospital-based case-control study in a family medicine outpatient setting. Because the identified hotspot area was in central Pingtung County, there was only one medium-sized local hospital, with 178 beds. In order to recruit COPD and chronic kidney disease (CKD) patients at the same time, we collaborated with only one family physician in the studied hospital. The family physician gave out flyers describing this survey to the patients who met our inclusion criteria. Then, the patients were free to decide whether to go to our survey site. Our interviewers introduced the background of the survey and gave one informed consent form to the patients and explained the contents. The patients did not need to sign the informed consent and could not withdraw from the study after finishing the questionnaire, because this questionnaire and the informed consent were totally anonymous, as explained in the informed consent form. When the patients finished the questionnaire, they were given a convenience store gift certificate worth NTD 100 (USD 3.20). 

In this study, the criteria for picking the studied area were twofold, including the highest relative risk in both genders, and the clusters of the estimating residuals after geographically weighted regression in our previous ecological study [4]. The first criterion focuses on the spatio-temporal aberrations of COPD mortality and the second focuses on the unexplained clustering areas after adjusting for known ecological factors. We then chose this area for further epidemiological investigation.

### 2.3. Mortality Calculation and Cluster Detection

The cause of death data for adults aged 40 and older at the township level from 2000 to 2012 were collected from the Ministry of Health and Welfare in Taiwan. Because of privacy concerns, townships with fewer than three deaths needed to be omitted. The same criterion was also applied when we computed the nationwide age-adjusted morality rate when deaths in specific age groups were fewer than three (*i.e.*, the number of deaths was then omitted). The population data were acquired from the Census database of the Ministry of the Interior. Two definitions of COPD deaths were used, including the International Classification of Diseases, Ninth Revision (ICD-9) codes before 2008 and ICD-10 codes after 2008. The ICD-9 codes included 490 (bronchitis, not specified as acute or chronic), 491 (chronic bronchitis), 492 (emphysema), and 496 (chronic airway obstruction, not elsewhere classified). The ICD-10 codes included J40 (bronchitis, not specified as acute or chronic), J41 (simple and mucopurulent chronic bronchitis), J43 (emphysema) and J44 (other chronic obstructive pulmonary disease). The direct age adjustment method was applied for nationwide age-adjusted mortality rates. The reference population was the year-2000 Taiwan population ≥40 years old. 

For cluster detection, we used Poisson-based space-time scan statistics [15] in SaTScan v.9.1.1 (http://www.satscan.org/) to detect the spatio-temporal clusters of crude COPD mortality from 2000 to 2012. All the 13 years’ mortality data for males and females were used, with a maximum cluster population size of 5% to minimize false clusters, and a maximum temporal window of one year to examine the clusters. The local risk maps were done by ArcGIS (ArcMap, version 10.2; ESRI Inc., Redlands, CA, USA). 

### 2.4. The Definition of Case and Control

After identifying one hotspot area, the research team collaborated with a family physician in one local hospital located in Pingtung County of southern Taiwan to identify cases aged ≥40 years old [5,16] with a clinical diagnosis of confirmed COPD with either ICD-9 or ICD-10 diagnosis, as listed in the previous section. For the control group, criteria for inclusion were age ≥40 years old and a clinical diagnosis of confirmed chronic kidney disease (CKD) with the ICD-9 code, 585 or ICD-10 code, N18. These two chronic diseases primarily strike the adult population, but affect different target organs and systems. COPD involves the respiratory system and CKD the excretory system; they also have different causes. The similar demographic factors were important for matching for the case-control study [17]. In this study, we found age, gender, BMI, and education had no significant differences at the baseline.

### 2.5. The Variables Collected by Questionnaire

We collected two types of data by questionnaire including demographic information and health history. The demographic information included gender, age, height, weight, village of current residence, primary village of residence in the past, education status, average monthly household income, whether or not ethnicity is aborigine, and occupation. The health history included “Are you a COPD patient?”, “Are you a CKD patient?”, “Have you ever been a smoker?”, “Have you ever consumed alcohol regularly?”, “Have you chewed betel nut before?”, “Have you burned charcoal or firewood in the house?”, and “Have you ever had a positive test result for tuberculosis (TB)?”.

### 2.6. Statistical Analysis and Spatial Visualization

We used logistic regression for analyzing the personal risk factors of COPD. The variables listed in the previous section are all included in the initial model. Then, we used a stepwise selection method based on the likelihood of selecting the statistically significant variables in the final model. We applied a chi-squared test, independent T-test and logistic regression with SPSS 20.0 (IBM Corp., Armonk, NY, USA). The results of local relative risk of COPD mortality and the spatial distribution of the cases and controls are plotted by ArcGIS. 

## 3. Results 

**Figure 1:**
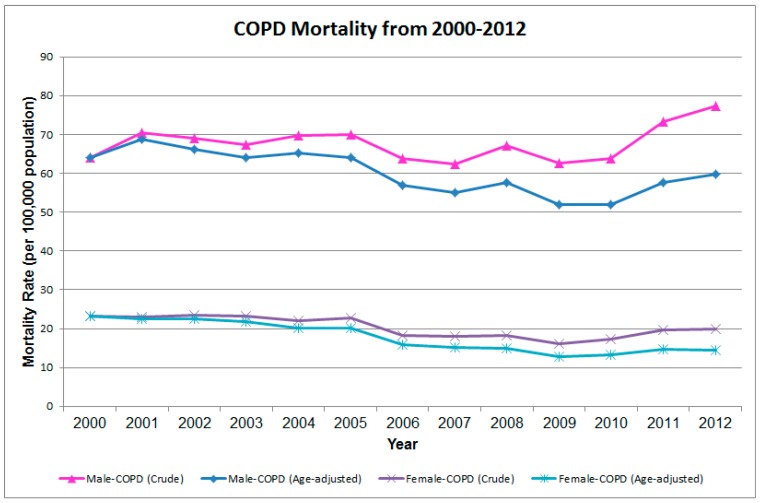
Temporal trend of crude and age-adjusted mortality (aged 40 years or older) of chronic obstructive pulmonary disease (COPD) in Taiwan from 2000–2012.

In Figure 1, the trend of COPD mortality for those aged 40 years or older can be clearly divided into two parts. From 2000 to 2009, the male COPD age-adjusted mortality declined from 64.13 per 100,000 population to 51.96 per 100,000 population, and the female COPD age-adjusted mortality also declined from 23.20 per 100,000 population to 12.81 per 100,000 population. However, the trend of mortality began to climb after 2009 in both genders. From 2010 to 2012, the male COPD age-adjusted mortality increased from 51.89 per 100,000 population to 59.67 per 100,000 population, and the female COPD age-adjusted mortality also increased from 13.12 per 100,000 population to 14.48 per 100,000 population.

**Figure 2:**
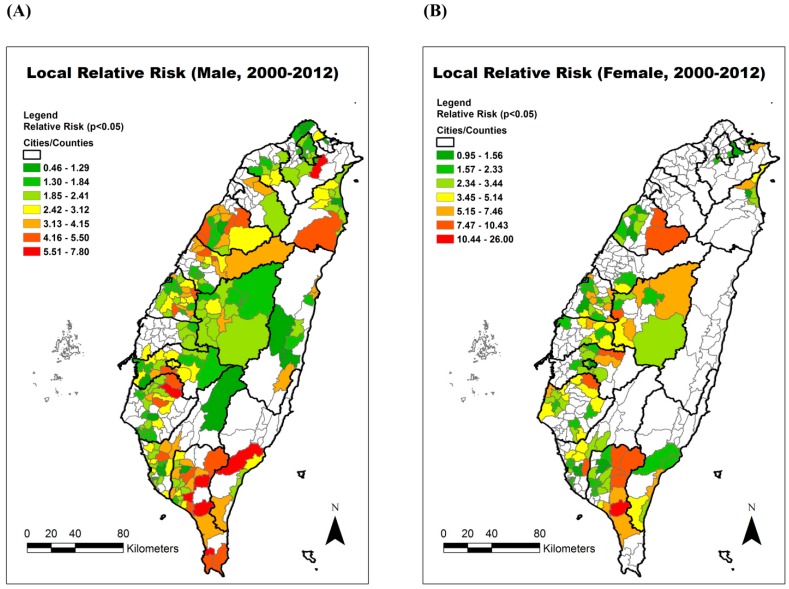
The estimated local relative risk of chronic obstructive pulmonary disease (COPD) in Taiwan from 2000–2012. (**A**) Male; (**B**) Female.

The spatio-temporal significant clusters (*p* < 0.05) are all displayed in color in Figure 2. The color classification represents the magnitude of the local relative risk. In both males and females, the highest relative risks of townships were found in Pingtung County of southern Taiwan. Thus, we chose one local hospital within this hotspot area to recruit participants. 

**Figure 3:**
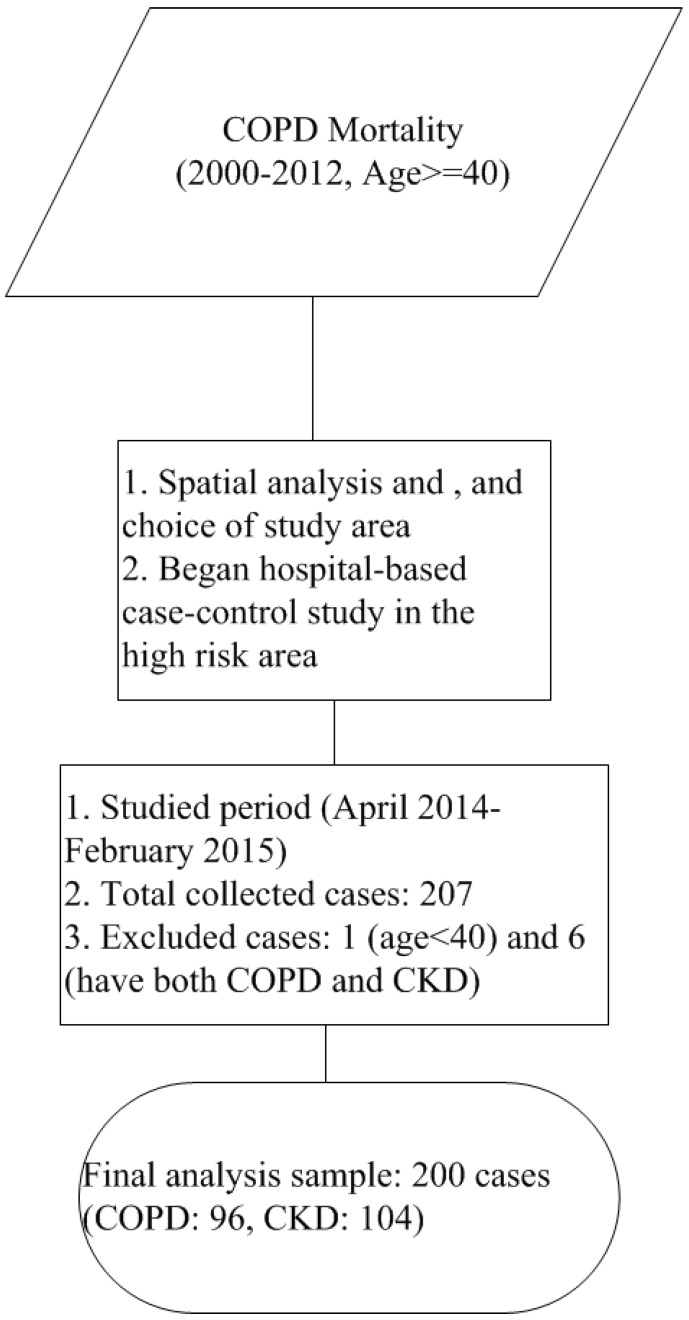
Recruitment flow chart

**Figure 4:**
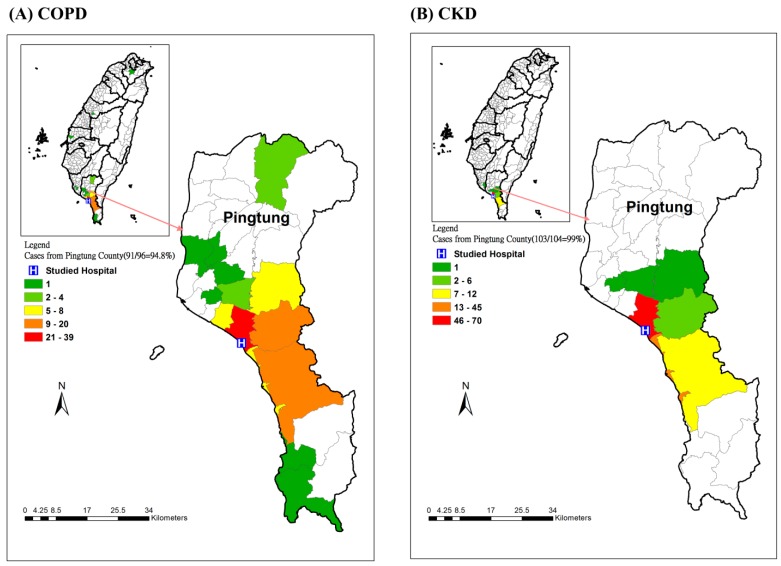
The spatial distribution of the patients in the case-control study (**A**) COPD; (**B**) CKD.

From April 2014 to February 2015, we successfully collected 207 questionnaires, and the family physician issued 133 flyers for COPD patients and 143 flyers for CKD patients. The response rate for COPD was 76.69% (102/133), and that for CKD was 73.43% (105/143). However, we excluded one patient aged less than 40 years old and six patients who had both COPD and CKD. After excluding those seven patients, the valid response rate for COPD patients was 72.18% (96/133), and that for CKD patients was 72.73% (104/143). The number of final included patients was 200, including 96 COPD patients and 104 CKD patients. The overall flowchart of the study is shown in Figure 3. The spatial distribution of the included patients is displayed in Figure 4. Figure 4A shows that 94.8% of COPD patients were from Pingtung County, and most of the cases were from the studied hospital’s surrounding townships. Figure 4B shows that 99% of CKD patients were from Pingtung County, and most were from the neighboring townships in Pingtung County.

From univariate analysis of the questionnaire (Table 1), we found that age and education are comparable between COPD and CKD groups. Although household income and occupation also showed non-significant difference between the two groups, the missing rate was too high in household income, and the housewives had a higher proportion in the COPD group (18.75%) than the CKD group (10.58%). Thus, we cannot make relevant inferences from these two factors. The percentage of aborigines in the COPD group is around 36.5%, which is much higher than that in the CKD group, 17.3% (*p* = 0.002). As for health history, we found that the TB history (*p* = 0.005) and smoking history (*p* = 0.002) are the significant risk factors for the patients with COPD. 

Then, we further used stepwise multivariate logistic regression to select the significant risk factors for COPD (Table 2). The results showed that the patients with smoking history had a significantly higher risk for developing COPD (adjusted odds ratios (AORs): 2.64, 95% CI: 1.46–4.76), and the patients who are aborigines also had significantly higher risk (AORs: 3.01, 95% CI: 1.52–5.93). Although TB history was significant in the univariate analysis, there were only seven cases in the COPD group, causing this coefficient estimation to not reach convergence. Thus, we also excluded the TB history from our final model. 

Smoking is a well-known risk factor for developing COPD. However, ethnicity might be correlated with some socio-economic or personal risk factors. Thus, we further stratified ethnicity, and observe the effects under it. Among the aboriginal group, the risk factors did not show statistical significance, which might be caused by the small sample size, only 53 respondents. But, the charcoal smoke exposure was higher in the aboriginal COPD group (57.1%) than in the non-aboriginal COPD group (21.3%). Among the non-aboriginal group, TB and smoking exposure were the two significant factors among COPD patients. In addition, we also analyze the interaction for smoking and charcoal fire exposure in the new Table 3 by logistic regression. The results show that the interaction term was not statistically significant (*p* > 0.05) in both the aboriginal group and non-aboriginal group. 

**Table 1:** The differences of demographic variables and risk factors between COPD and CKD patients.

Types of Variables	Variables		COPD	CKD	*P*-Value
Demographic factors	Age	40–64	27	33	0.58
		≥65	69	71	
	Gender	Male	63	59	0.11
		Female	31	45	
		Missing	2	0	
	Aborigine	Yes	35	18	0.002 *
		No	61	86	
	Education (completed)	Illiterate	20	25	0.41
		Elementary school and junior high school	62	58	
		High school and above	14	21	
	BMI	(kg/m^2^)	25.28 (SD:4.30)	26.09 (SD:3.59)	0.15
Socioeconomic status	Household Income (Currency: 1USD = 31TWD)	≤645 USD	44	38	0.41
		>645 USD and ≤3871 USD	12	16	
		Unknown or uncertain	40	50	
	Occupation	Farming, fishing, and forestry occupations	40	42	0.24
		Workers	24	24	
		Service occupations	2	10	
		Housewives	18	11	
		Retired	3	4	
		Office and administrative support occupations	6	6	
		Other or unemployed	3	6	
		Missing	0	1	
Personal risk factors	TB history	Yes	7	0	0.005 *
		No	89	104	
	Smoking history	Yes	55	37	0.002 *
		No	41	67	
	Drinking	Yes	39	29	0.06
		No	57	75	
	Betel nut	Yes	27	18	0.07
		No	69	86	
	Burned charcoal or wood	Yes and mostly used indoors	9	12	0.11
		Yes and mostly used outdoors	24	14	
		No	63	78	

* *p* < 0.05

**Table 2:** The significant risk factors for COPD.

Variables		Adjusted Odds Ratios (AORs)	95% CI of AORs	
			LCI	UCI
Smoking history	No	Reference		
	Yes	2.64	1.46	4.76
Aborigine	No	Reference		
	Yes	3.01	1.52	5.93

**Table 3:** The differences of personal risk factors between COPD and CKD patients stratified by ethnicity.

Personal Risk Factors		Aborigines			Non-Aborigines		
		COPD	CKD	*P*-Value	COPD	CKD	*P*-Value
TB history	Yes	3	0	0.28	4	0	0.028 *
	No	32	18		57	86	
Smoking history	Yes	16	7	0.64	39	30	0.001 *
	No	19	11		22	56	
Drinking	Yes	16	10	0.5	23	19	0.039 *
	No	19	8		38	67	
Betel nut	Yes	17	8	0.78	10	10	0.41
	No	18	10		51	76	
Burned charcoal wood	Yes and mostly used indoors	5	1	0.39	4	11	0.32
	Yes and mostly used outdoors	15	6		9	8	
	No	15	11		48	67	

* *p* < 0.05

## 4. Discussion 

This study analyzed COPD mortality over a period of 13 years from both temporal and spatial perspectives. In both genders, the crude and age-adjusted COPD mortalities rose gradually after 2009. The reason might be the increasing elderly population in Taiwan’s society. The percentage of elderly (age ≥ 65 years old) in the overall population increased from 8.62% in 2000 to 11.15% in 2012 (http://sowf.moi.gov.tw/stat/year/list.htm). The aging lung function also leads to developing COPD [18]. The spatial distribution of the highest local relative risk is clustered in Pingtung County, which is the southernmost county in Taiwan. Therefore, we tried to elucidate the local risk factors for developing COPD within the hotspot area. Then, we conducted the small case-control study in one local hospital. 

**Figure A1:**
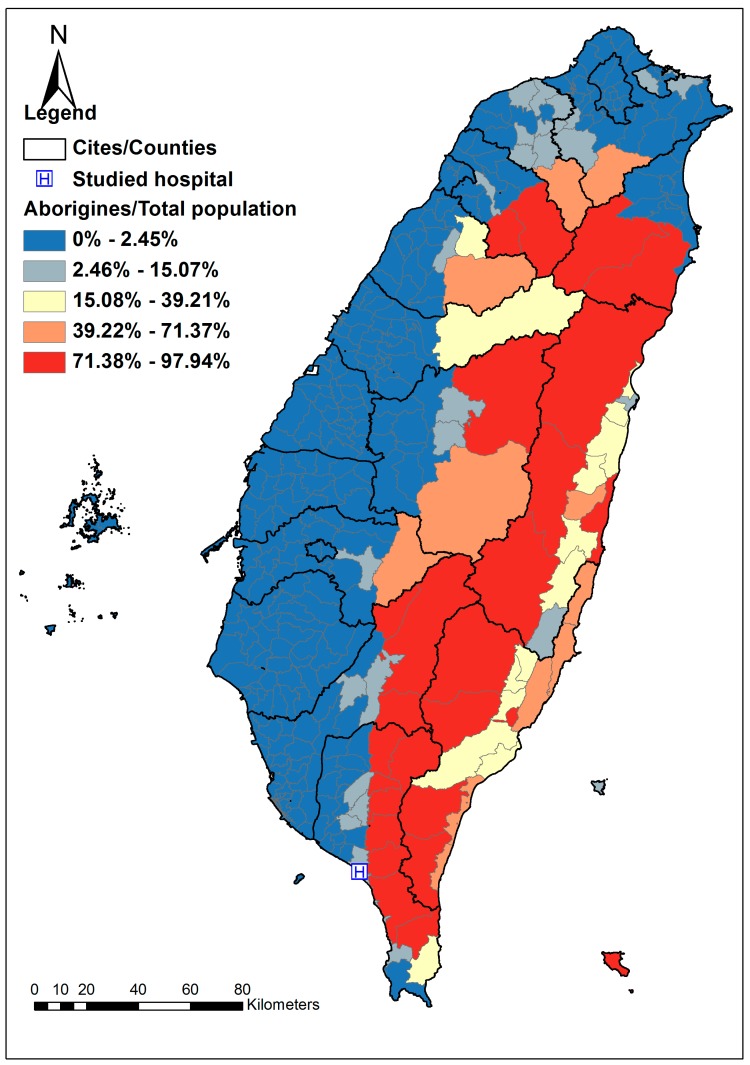
The percentage of aborigines in Taiwan in 2012.

**Figure A2:**
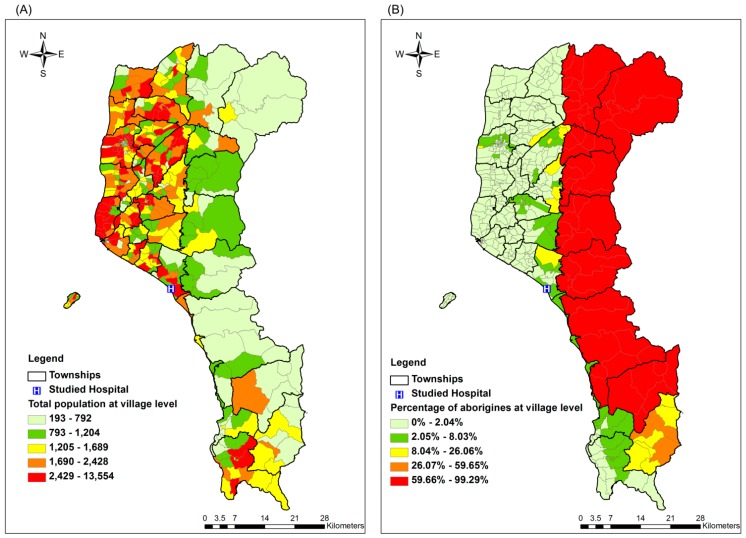
The spatial distribution of the total population in Pingtung county, and percentage of aborigines at village level.

The findings showed that risk factors, including smoking history and aborigine status, are consistent between our previous ecological studies [4] and this case-control study. Smoking is a well-known risk factor for COPD [6,7] and we also confirmed this correlation at the ecological and individual level. The other risk factor is being an aborigine. From an ecological view, the eastern and southern townships of the studied hospital had populations with over 90% aborigines (Figure A1). A similar phenomenon is also found in Alberta (Canada) [19]. They found that three aboriginal groups had a higher prevalence and incidence of COPD compared to a non-aboriginal cohort even after controlling for other demographical factors and socio-economic status (SES). In Taiwan, lower average SES [20], lower medical access [21], and a higher smoking rate [22] have been found among the aboriginal population. In Figure A2, we can see that the aborigines live chiefly in the eastern side of Pingtung County, which is a mountainous area. The residents of townships surrounding the studied hospital are more than 60% aborigines, while the township where the hospital is located has a lower percentage of aborigines but higher population than surrounding townships. In addition, Pingtung County had the highest incidence of TB in Taiwan in 2012 (95.1 per 100,000) and the fourth highest TB mortality (5.5 per 100,000) [23]. Although TB history cannot reach convergence in the multivariate logistic regression, a significant difference was observed in the univariate comparison. Previous studies have showed that delayed diagnosis and treatment of TB might increase the risk of developing COPD [24], and also some studies have showed that COPD patients will have higher risk of developing active TB [25]. This evidence reflects the strong correlation between TB and COPD. Thus, the risk from TB cannot be ignored, and more patients need to be recruited to validate the correlation in the future. 

The other important risk factor was the exposure to biomass smoke. Burning charcoal or wood for cooking and keeping warm is very traditional for some elderly people. If we consider the behaviors of burning charcoal or wood indoors and outdoors together, there is a higher percentage among COPD patients (34.4%) compared to CKD patients (25%), though this did not reach statistical significance. In Africa, biomass smoke exposure has been correlated to the incidence of COPD [26].

Among our studied patients, most of the baseline demographic variables showed no difference between COPD and CKD patients. However, overall, the participating patients had lower educational status and lower household income, and were in farming, fishing and forest, or worker occupations. COPD and CKD are two diseases with high disease burdens in Taiwan. The characteristics of the patients imply possible health inequities between the urban and rural areas in Taiwan. 

In addition, most of the patients are from the townships surrounding the studied hospital. Because Taiwan has 99% coverage of national health insurance, people can choose to visit either a clinic or hospital, with a very small payment for either one. In Taiwan, the referral system is very weak. People are able to choose their healthcare providers freely [27]. Figure 4A shows that there was only one case each from a northern township and southern township. The major sources of cases and controls are from central Pingtung County, which only has one hospital.

There were five limitations in this study. The first one is the cell-size limitation when using the cause of death database. Due to privacy concerns, we could not compute aggregations from fewer than three deaths. Thus, we underestimate the true mortality in each township. In addition, we also could not compute age-adjusted mortality by township level because the number of deaths was low, and we could not differentiate into many age groups and keep cell numbers larger than two. Thus, we were only able to calculate annual nationwide age-adjusted COPD mortality. The second limitation is the recruitment of the patients. We calculated that the sample size should be larger than 200 to reach the statistical power of 80%. Because of our limited study budget, we could only recruit cases and controls from one family physician, but some patients go directly to either the chest medicine department or nephrology department. Thus, the recruiting process lasted around 11 months. Because of the sample size and missing values, some risk factors such as income did not have enough power to explain, and some, such as occupation, were not distributed evenly, which also might affect the model’s power. However, the findings in this study can provide the clues for further comprehensive survey for the selected townships. The third limitation is the cross-sectional design. We could not examine the temporal relationship between the risk factors and the disease. Thus, we were only able to show correlations between the risk factors and COPD. The fourth limitation is the selection of our control group. In this study, we selected another important chronic disease in Taiwan, CKD. Although smoking might also elevate the risk of developing CKD [28], smoking was still a significant risk factor, the role of which might be underestimated in this study. For other demographic variables, these two groups are comparable. The fifth limitation is from the self-report questionnaire. The disease status was diagnosed by the family physician, then the qualified respondents could get the flyer and decide whether to go to our survey site. However, the risk factors listed on the questionnaire were all self-reported, and underreporting may have occurred. Take the TB history as an example. Because TB is an infectious disease, the self-reporting of this disease might lead to underreporting due to the stigma associated with it. Thus, the actual effect of TB on COPD might also be underestimated here.

## 5. Conclusions 

This study demonstrated the integration of the ecological study and case-control study methods for investigating the risk factors for COPD. The temporal trend showed that COPD mortality was climbing after 2009 in Taiwan. There was a spatio-temporal cluster of higher relative risk of COPD mortality in southern Taiwan. Smoking and aboriginal ethnicity were two important risk factors correlated with COPD. Although we cannot identify the specific significant personal factors among the aboriginal group from the stratification results, a history of TB and exposure to biomass smoke were two possible important risk factors we cannot ignore, and a large-scale survey is needed for validating the correlations. This two-stage approach might be beneficial to examine and cross-validate the findings from aggregation to an individual scale, and can be easily extended to other chronic diseases.
